# Crumpling of thin sheets as a basis for creating mechanical metamaterials

**DOI:** 10.1039/c8ra07565d

**Published:** 2019-02-11

**Authors:** M. C. Fokker, S. Janbaz, A. A. Zadpoor

**Affiliations:** Department of Biomechanical Engineering, Faculty of Mechanical, Maritime and Materials Engineering, Delft University of Technology Mekelweg 2 Delft 2628CD The Netherlands marike.fokker@gmail.com

## Abstract

Crumpled thin sheets exhibit extraordinary characteristics such as a high strength combined with a low volume ratio. This review focuses on the physics of crumpled thin sheets, including the crumpling mechanics, crumpling methods, and the mechanical behavior of crumpled thin sheets. Most of the physical and mechanical properties of crumpled thin sheets change with the compaction ratio, which creates the opportunity to obtain the properties that are needed for a specific application simply by changing the compaction ratio. This also enables obtaining unusual combinations of material properties, which cannot be easily found in nature. Furthermore, crumpling starts from a flat surface, which could first be decorated with (nano-) patterns or functionalized through other surface treatment techniques, many of which are only applicable to flat surfaces. Ultimately, the crumpling of thin sheets could be used for creating disordered mechanical metamaterials, which are less sensitive to geometric imperfections compared to ordered designs of mechanical metamaterials that are based, for example, on origami or lattice structures.

## Abstract 2

Random formation of layers and ridges through the crumpling of a flat matter can form a robust mechanical metamaterial.

## Graphical abstract

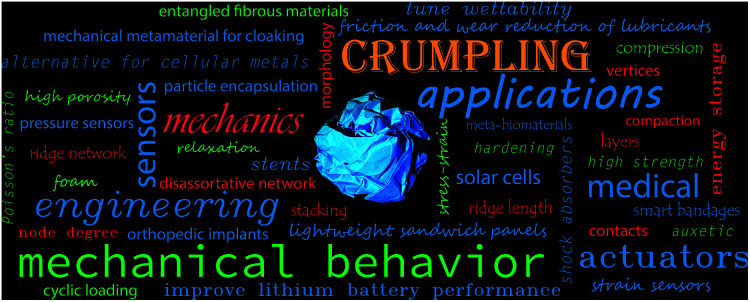

## 1. Introduction

Crumpling, the process of networking between vertices and ridges in thin sheets, is frequently observed in nature. Examples are flower buds,^1^ DNA packing in viral capsids,^2,3^ the buckling of the crust of the earth,^4^ and the morphogenesis of the brain cortex.^5^ Although crumpling is a random process, certain relationships between the geometry and force-related parameters could be established under well-defined conditions.^6–11^ Interestingly, the correlation between the thickness of the cortex and the surface area in mammalian brains has been found to be in agreement with those found for paper-based crumpled balls.^5,6^ A crumpled paper ball, which is often studied as a model of crumpling,^12–14^ could be considered as a folding-based approach to create disordered three-dimensional metamaterials^15,16^ out of a flat sheet. Understanding the physics of crumpling may therefore be useful for designing crumpling-based metamaterials.^6,17^ The transformation from two to three dimensions could facilitate surface functionalization given the fact that many surface functionalization techniques such as nanolithography^18–20^ or electron-beam induced deposition^21^ are only applicable to flat materials. A well-controlled crumpling process could guarantee proper spatial distribution of properties within the structure and produce the details of the planned microstructures within the final three-dimensional construct.^22^

In this paper, we review the existing literature on the crumpling of planar materials with the aim of exploring the possibilities and providing strategies for fabrication of 3D porous materials. In the first section, we describe the methods that could be used to create crumpling-based 3D materials. Then, the different factors influencing the folding mechanics are described. The relationships between the applied forces and deformations as well as the morphological characteristics (*e.g.* density, porosity, surface curvature, *etc.*) and the related folding networks are discussed as well. Furthermore, the relationships between the mechanical properties of crumpled structures such as stiffness, strength, and relaxation and the underlying physics are addressed. Finally, a range of potential applications and strategies for designing advanced metamaterials are proposed.

## 2. Crumpling methods

**Fig. 1:**
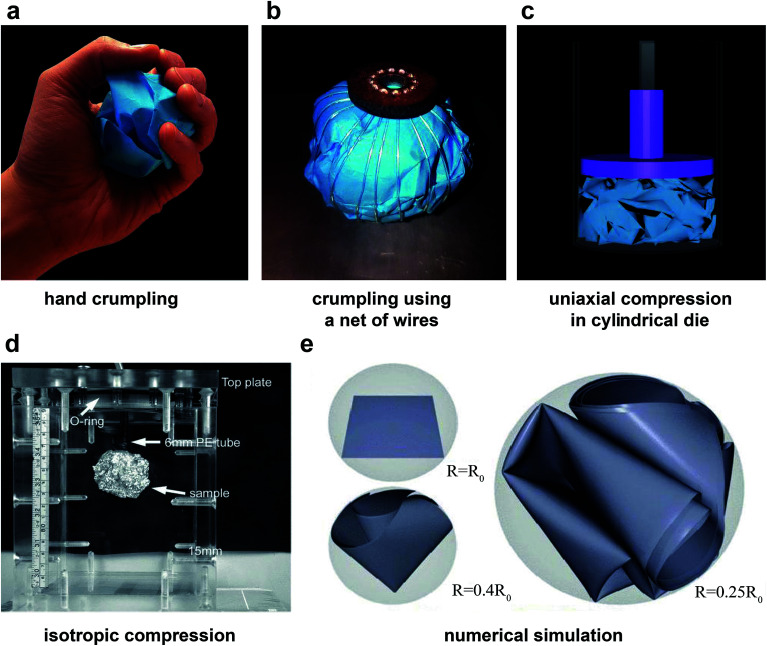
Crumpling methods with (a) crumpling by hand, (b) crumpling with the use of a net of wires evenly distributed over the surface of the ball, and (c) crumpling in a cylindrical die with axial compression. (d) Isotropic compaction of aluminum crumpled balls using ambient pressure.^29^ (e) Numerical simulation of crumpling in a spherical shell.^34^

A crumpled structure could be obtained using numerous crumpling methods of which crumpling by hand (Fig. 1a) seems to be the most commonly used technique.^9,12,23–28^ Hand crumpling could be done in different ways. Three different techniques were compared by Plouraboué and Roux.^28^ In the first method, crumpling starts from the corner of the sheet and gradually includes more surface until the whole sheet has been crumpled. The second technique is the same as the first method with the exception that it starts from the center of the sheet. In the last method, a number of large folds are initially introduced to the sheet. The crumpling then proceeds by compressing the loose ball further. No significant differences were found in the geometry of the unfolded sheet or the size of the crumpled ball.^28^ Although crumpling by hand is the easiest way to crumple a thin sheet^29^ and it can produce a very compact ball,^23^ it may not be the most well-defined way, as it is highly diverse and irreproducible.^23,29^ The applied force is different between different individuals, it cannot be measured, and is not well-regulated or standardized.^29^ Furthermore, the size of the hand of the person who crumples the thin sheet introduces a length scale, which cannot be controlled.^23^

The method most similar to hand-crumpling is perhaps crumpling in a 3D sphere, which could change in radius. This method is often used in simulations and numerical models of the crumpling of thin sheets (Fig. 1e).^13,30–34^ However, this method is much more difficult to apply in practice.

Habibi *et al.*^6^ and Mirzaali *et al.*^17^ used a net of wires distributed equally over the surface of a loosely crumpled ball (Fig. 1b). The wires go all through a hole in the bottom plate and are connected to a weight, which causes the wires to tighten equally and decrease the radius of the sphere they create.

Lin *et al.*^29^ used ambient pressure on a loosely crumpled ball to compress it further (Fig. 1d). They found that the force that was needed to crumple aluminum and high-density polyethylene (HDPE) sheets with ambient pressure was much higher than in crumpling by hand. In addition, a much lower crumpling ratio can be reached with crumpling by ambient pressure as compared to crumpling by hand.^29^ In the most confined state, the crumpled ball they created contained only 7% body fraction, while this could be up to 25% in hand-crumpled specimens.

Another approach to crumple a sheet within a sphere is to keep the sphere the same size while enlarging the thin sheet. Aharoni and Sharon^10^ used temperature-responsive elastic sheets in a glass sphere filled with water in which the swelling of the sheets could be controlled by controlling the temperature of the water. This technique could only be applied to specific materials. Spherical crumpling of thin sheets is challenging in practice, which is why it has not been widely used.

A frequently used crumpling technique is application of a cylindrical die (Fig. 1c),^7,11,23,35–37^ which is known to be well reproducible.^36,37^ Cambou and Menon^35^ compressed polydimethylsiloxane (PDMS) sheets either radially, by decreasing the radius of the cylindrical die, or axially, by reducing the height within the cylindrical die with a flat piston. They showed that using a cylindrical die to crumple a sheet causes vertical alignment near the top and bottom plate regardless of the confinement method (axially or radially). Cottrino *et al.*^36^ observed similar behavior when crumpling aluminum sheets in a cylindrical die through axial confinement. This alignment is much stronger in cylindrical confinement as compared to spherical confinement.^35^ The cylindrical die is also used to prepare the samples such that they could undergo compression tests.^36,37^

To combine the strengths of several methods and minimize the limitations, the methods are sometimes combined. An example is hand-crumpling combined with compression by a piston^38^ or squeezing by a tip-tweezer,^8^ where a loose ball is formed by hand and further compression is accomplished using the other technique.

Apart from the method used to crumple a thin sheet, the crumpling rate seems to influence the characteristics of final structure. In aluminum foils, it has been shown that the stress–strain behavior depends on the loading rate^38^ particularly when compressed in a simple compression test.^36^ Simulations of a rectangular, elastic sheet crumpled in a spherical shell show that configurations with low elastic energy are more likely to appear during slow deformation.^34^ Tallinen *et al.*^34^ discovered that when frictionless elastic sheets are crumpled slowly, two different final modes could be achieved: a highly random crumpled ball with high deformation energy and entropy or a crumpled ball with symmetric folds showing a low entropy and deformation energy. Both modes were found to appear equally frequently, implying that there is a competition amongst states of low energy and high entropy.^34^

Additionally, simulations on graphyne nano sheets crumpled in a spherical shell^32^ exhibit the importance of the crumpling rate as well. At slow crumpling rates, high symmetry is usually detected in the folding, while high crumpling rates result in a much more random folding pattern and show an approximately constant increase of potential energy during the confinement.^32^ This indicates that higher stress levels are reached within the sheet when crumpled under a higher crumpling rate.^32^ Moreover, Becton *et al.*^32^ discovered that the temperature and the initial shape of the graphyne nano-sheet affect the geometry of the crumpled structure. At low temperatures, the packing of graphene is more rigid and it shows a higher tendency to unfold when the confinement pressure is released.^32^ However, the overall behavior does not differ significantly. In general, the only difference is an increase in flexibility with temperature, initiating a higher propensity for abnormal crumpling modes.^32^ The geometry of the graphyne nano sheet particularly affects the initial crumpling behavior.^32^ A circular sheet will first form a horn before self-adhesion causes the sheet to roll up, while in a square sheet the corners will fold towards the diagonal opposite corners and then buckle in the middle until self-adhesion occurs and the sheet will form an indeterminately folded sheet.^32^ In a triangular sheet, two corners will fold in the same direction, while the third corner will fold in the opposite direction.^32^ Notwithstanding those differences within the initial crumpling, the hardness and the bulk modulus of the triangular and square sheets were found to be within 10% of the hardness and the bulk modulus of the circular sheets, suggesting that the final crumpled structures of all three geometries are similar.^32^

## 3. Mechanics of crumpling

Crumpled papers have been widely studied to reveal the physical principles governing the process of crumpling flat thin materials.^12,14,23–25,27,39,40^ A flat piece of paper exhibits different patterns of buckling depending on the loading conditions. External load distribution as well as evolution in the boundary conditions of a confined paper could determine the trend of formation of wrinkled patterns. Successive forces result in elastoplastic deformations, which are followed by higher levels of consecutive folding patterns. Sharp folds will appear as a result of compaction, thereby dissipating energy. Once a certain level of compaction has been achieved, a network of self-contacts forms within the structure, limiting further changes in the dimensions of the crumpled sheet. That is despite the fact that a significant part of the structure is still air at this stage.^7,8,11,30^

### 3.1. Compaction

**Fig. 2:**
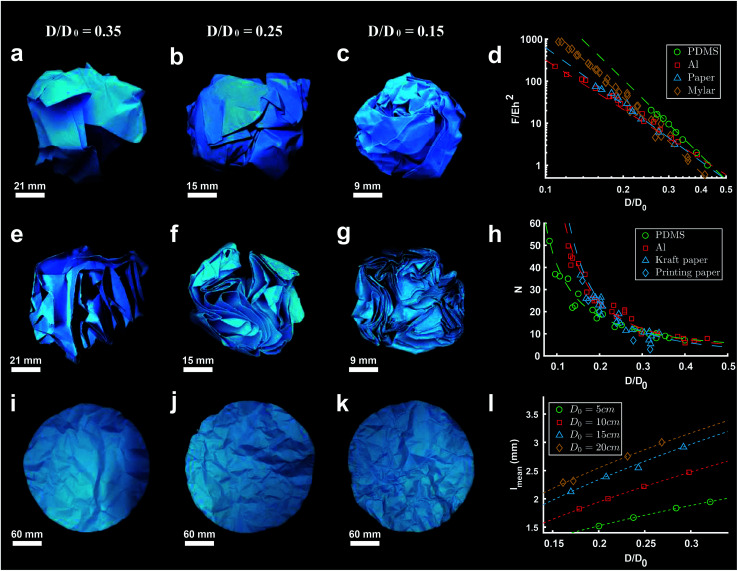
(a–c) Hand-crumpled paper balls with a compaction ratio (*D*/*D*_0_) of 0.35, 0.25, and 0.15 respectively. (d) Dimensionless crumpling force (*F*/*Eh*^2^) as a function of compaction ratio (*D*/*D*_0_) in crumpled sheets of PDMS (green), aluminum (red), paper (blue) and mylar (yellow), fitted by eqn (2) with *β* is 6.0, 5.9, 4.5 and 3.9 for PDMS, mylar, printing paper and aluminum respectively.^6^ (e–g) Cross sections of the crumpled paper balls shown in (a–c), showing the layers created during the crumpling process. (h) Number of layers (*N*) as a function of compaction ratio (*D*/*D*_0_) for crumpled PDMS (green), aluminum (red) and paper (blue), based on findings of Habibi *et al.*^6^ and Deboeuf *et al.*,^7^ fitted by eqn (5) with *γ* is 1.2, 1.7 and 2 for aluminum, PDMS and paper respectively. (i–k) Unfolded crumpled paper balls with compaction ratios (*D*/*D*_0_) corresponding to the crumpled balls in (a–c), revealing the ridge network created during crumpling. (l) Correlation between average ridge length (*l*_mean_) and compaction ratio (*D*/*D*_0_) in crumpled paper, based on the results of Balankin *et al.*,^39^ showing the mean length of ridges scales with the initial size of the sheet and the compaction ratio (*D*/*D*_0_).

Simulations of crumpling thin circular sheets in a sphere show that when the compaction force exceeds a threshold, the flat sheet will first start to deform into a developable cone (d-cone).^30^ As the force increases, the material will start to buckle and ridges and layers form, thereby creating a crumpled structure (Fig. 2).^6,35^ The resistance to further compaction develops with the degree of compaction even though the compacted structure still contains a significant amount of air.^7–9,11,30^ This is probably due to the morphology of crumpled structures, which is affected by various parameters such as the initial size of the flat sheet, the magnitude of the confinement force, and the confinement boundaries.^6–11,29^

Crumpling of aluminum foils and high-density polyethylene (HDPE) films under ambient pressure has shown a power-law correlation between the compaction force (*F*) and the final ball size (*R*).^29^ This relationship could be expressed as:^29^

1 *R* ∝ *R*_0_^*ν*^*F*^−*α*^

where *R*_0_ is the initial radius of the crumpled material and *ν* and *α* are universal values respectively equal to 1/4 and 4/5 for sheets who cannot cross themselves.^29^ The behavior described through this relationship sheet is otherwise known as the self-avoidant behavior of a thin sheet.^30^ This behavior becomes more prominent in more advanced stages of compaction.^30^

Similar power laws relating the compaction ratio to the compaction force have been found in other studies.^6–11^ Habibi *et al.*^6^ crumpled polydimethylsiloxane (PDMS) (thickness *t* = 1 mm), Mylar (*t* = 75, 36, 23 and 19 μm), regular printing paper (*t* = 100 μm), and aluminum foils (*t* = 20 and 8 μm) using a net of wires, distributed homogeneously over the external area of a loosely crumpled material (Fig. 1b). All materials showed a power-law behavior (Fig. 2d) which could be expressed as:2
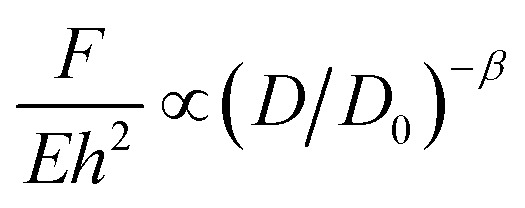
where *F*, *E*, *h*, *D*, *D*_0_, and *β* are respectively the compaction force, elastic modulus, thickness, diameter of the crumpled ball, initial diameter of the ball, and a plasticity-dependent exponent. *Eh*^2^ corresponds to the force needed to make a single fold in the material.^6^ Dissimilarity in the plasticity of studied materials shows a decrease in the exponent *β*, as the degree of plasticity increases.^6^

Simulation results indicate similar power-law relationships between the compaction force and the dimensions of the compacted geometry. In particular, Vliegenthart and Gompper^30^ studied the confinement of a 2D sheet in a 3D sphere through simulations. They found that when the compaction force exceeds a certain threshold, the correlation between the radius of the crumpled material and the compaction force obeys a power-law function, which could be expressed as:^30^3
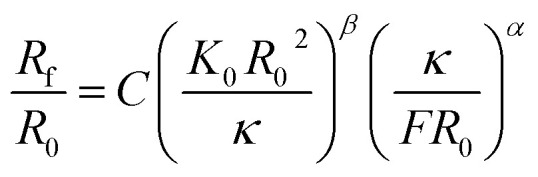
with *R*_f_, *R*_0_, *C*, *K*_0_, *κ* and *F* representing the radius of the crumpled sheet, the initial radius of the flat sheet, a constant, the two-dimensional Young's modulus (
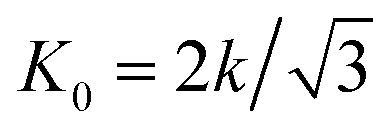
, where *k* is the spring constant), bending rigidity, and the compaction force, respectively. The exponent *β* was found to be 0.06 and the exponent *α* to be 0.25 for self-avoiding sheets. The comparison between self-avoidant sheets and phantom sheets (sheets not obeying the self-avoiding requirement) shows that the *α*-exponent in phantom sheets is 0.38, which implies that phantom sheets are more easily compacted.^30^ In an experimental study,^29^ the value of the *α*-exponent was found to be lower for aluminum (0.195 ± 0.008) and HDPE foils (0.065 ± 0.002) crumpled under ambient pressure as compared to the computationally predicted value of 0.25. This suggests that *α* is probably dependent on other parameters such as the plasticity of the material as well, thereby supporting the findings of Habibi *et al.*^6^ Experiments show that the effects of sheet thickness on the *α*-exponent is negligible.^29^

At higher levels of compaction, the resistance of crumpled materials increases drastically,^29,40^ which is referred to as the locking state and corresponds to an exponential function.^29^ The critical force which leads to this state is determined by the thickness of the sheet and the type of material.^29^

### 3.2. Morphology

The final configuration of a crumpled sheet exhibits interesting geometrical features such as the number of layers, random morphology, stacking of layers, scattering of the two different sheet sides over the surface of the ball, and the probability of contact between the different sides of the sheet. Balankin *et al.*^25^ studied the distribution of both sheet sides over the surface of a ball in hand-crumpled paper balls. They found that if the ratio between the thickness and the initial size of the paper sheet is smaller than a critical value, one side of the sheet is more dominantly present at the crust of the ball. When the thickness to sheet size ratio exceeds this threshold, both sides of the paper sheet are statistically equally present at the ball surface.^25^ The threshold could be determined as:^25^

4 *t*/*L*_C_ = 4 × 10^−5^

where *t* and *L*_C_ stand for the thickness of the paper sheet and the critical sheet size, respectively.

Another interesting phenomenon that occurs during crumpling is the formation of various layers.^6,7,9,29,41^ Research in aluminum sheets crumpled by hand^41^ as well as aluminum and HDPE sheets crumpled under ambient pressure^29^ show that many layers are created in the initial steps of the crumpling process. This is most likely due to the high degree of freedom in space, allowing the material to form new layers.^29,41^ However, when the material becomes denser, the possibility to form new layers becomes limited, resulting in a lower amount of newly formed layers.^29,41^ Even further confinement only enables the material to buckle and surge, as there is no space left to form new layers.^29,41^Fig. 2e–g show the cross-sections of the corresponding hand-crumpled paper balls in Fig. 2a–c, illustrating that the number of layers increases with increasing compaction.

The correlation between the number of layers and the number of vertices in crumpling aluminum foil and HDPE sheets under ambient pressure has been also studied.^29^ It shows that as the number of vertices increases, the number of layers decreases.^29^

Additionally, Habibi *et al.*^6^ found a power-law relationship between the size of the crumpled ball and the number of layers (*N*) in spherically crumpled PDMS, Mylar, paper, and aluminum foils:

5 *N* ∝ (*D*/*D*_0_)^−*γ*^

where *D*, *D*_0_ and *γ* are respectively the diameter of the crumpled ball, the initial diameter, and a scaling parameter. Fitting eqn (5) to the data found for PDMS and aluminum foils resulted in *γ* = 1.7 for PDMS and *γ* = 1.2 for aluminum foil (Fig. 2h),^6^ suggesting that the exponent *γ* changes slightly when the plasticity of the material changes. Deboeuf *et al.*^7^ found a similar relationship in hand-crumpled paper balls (*t* = 10 μm) with a *γ* value of 2 (Fig. 2h). This exact relationship was found in three dimensional folded paper as well,^7^ showing that, at least in paper, the geometry of a crumpled sheet is similar to that of three dimensional folded sheets.^7^ Habibi *et al.*^6^ discovered a linear relationship between the plasticity-dependent exponent *β* in eqn (2) and the exponent *γ* in eqn (5), indicating that this relationship holds independent from the material properties, but is, instead, dependent on the crumpling method.^6^ Furthermore, the number of layers could be affected by the plasticity of the material.^6^ This is due to the fact that irreversible wrinkles will occur in materials with high plasticity, which causes shrinkage of the effective area and will lead to a smaller number of layers than what is expected at a certain degree of compaction.^6^

The layers that originated during the crumpling process have the tendency to stack.^9^ Research in hand-crumpled aluminum sheets with a volume fraction of 8.5%^9^ shows more stacking in the outer layers of the crumpled ball as compared to the center of the ball. Additionally, higher numbers of layers are more often found in crumpled balls with a higher compaction, starting from the outer side and developing towards the core with growing compaction.^9^ This corresponds to the findings of Lin *et al.*^41^ demonstrating the mass tends to accrue at the crust of the ball in loose compacted aluminum balls. However, they state that the mass distribution becomes more homogeneous when the compaction grows denser. In crumpled aluminum samples compressed in a cylindrical die, the volume fraction is homogeneously distributed over the height but is nonhomogeneous over the diameter.^36^ Interestingly, the density decreases near the edges in those samples^36^ in contrast to hand-crumpled paper balls. Cottrino *et al.*^36^ found that the layers formed in crumpled aluminum foils tend to orient in a direction perpendicular to the compaction force. Furthermore, they found an increase in the number of contact points with the degree of compaction.^36^ The stacking behavior in crumpled structures therefore seems to depend on the crumpling technique.

Simulation of self-avoidant and phantom sheets crumpled in a three-dimensional sphere show that the differences between self-avoidant and phantom sheets are mainly caused by self-contacts.^30^ In loose compacted balls, only a few line-like contacts are present. The number of line-like contacts, however, grows with the compaction ratio as well with the two-dimensional contact area.^30^ The number of two-dimensional contact regions expands with further compaction and in highly compacted crumpled balls, the contacts mainly consist of two-dimensional contact regions rather than line-like contacts.^30^ Interestingly, ridges compressing on flat areas are rarely found.^30^ Another study, simulating a crumpled ball in one dimension, has also shown numerous self-contacts in high confinement.^40^

In hand-crumpled paper balls compressed by a constant force, the size and mass of the ball have been found to be correlated according to a fractal scaling law:^24,25^

6 *M* ∝ *R*^*D*^

where *M*, *R*, and *D* are the mass of the sheet, diameter of the crumpled ball, and the global fractal dimension of the set, respectively.^24,25^ The global fractal dimension *D* for elastic sheets is expected to be universal, if the ball diameter is much larger than the sheet thickness but smaller than the initial length of the sheet.^13,25,30^ In elastoplastic sheets, the global fractal dimension *D* has been found to be dependent on the properties of the material and the thickness of the sheet.^24,25,42,43^

### 3.3. Ridge network

In the early stages of compacting a flat material, buckles and wrinkles will arise.^44^ With further compaction, the amplitude of those wrinkles will grow and the wrinkles will transfer into sharp ridges and vertices.^31,44^ Eventually, plastic deformation will cause those ridges and peaks to be permanent and be visible even when the sheet is stretched out again (Fig. 2i–k). This visible network of ridges have been studied by several groups before.^7,12,14,24,39^ Balankin *et al.*^39^ determined the number of ridges in hand-crumpled paper balls by unfolding the crumpled paper balls and marking the ridges with a pencil. They discovered that the number of ridges scales with the size of the sheet and the compaction ratio. The mean ridge length was also found to be dependent on the initial size of the sheet and the compaction ratio (Fig. 2l).^39^ Andresen and Hansen^12^ used unfolded paper balls to examine the ridge network. They stretched the unfolded balls of paper to a standard size, secured the paper to an aluminum plate and measured the distance to the sheet using a laser. They found that the number of ridges and vertices not only depend on the size of the sheet and the compaction ratio but it is also dependent on the thickness of the sheet. This is confirmed by Dierking and Archer^45^ in their study on crumpled polymer films. They also found that thick polymer films display a higher number of long ridges as compared to thinner polymer films. Additionally, simulations of two-dimensional self-avoidant and phantom sheets confined in a three-dimensional sphere^30^ have shown a much larger number of ridges in a self-avoidant sheet. While the number of large ridges in phantom sheets has been found to be similar to the number of large ridges in self-avoidant sheets, the number of short ridges were much larger in self-avoidant sheets.^30^ Furthermore, they show that the increase in the number of ridges with the compaction ratio is much slower for phantom sheets than in self-avoidant sheets. Micro-computed tomography (micro-CT) analysis of hand-crumpled aluminum balls (compaction ratio of 8.5%)^9^ has shown that 37% of the surface points of the sheet are associated with folds and 4% with vertices. The same study also shows that the ridges are homogeneously distributed throughout the structure.

A laser and a CCD camera were used in another work to study ridge networks in flattened hand-crumpled paper balls.^14^ In this approach, the intersection of the vertical laser beam with the surface of de-crumpled paper is captured. Using this technique, it was found that loose compaction results in the formation of large ridges. After further compaction, those large ridges are randomly split into smaller ridges of various length. Blair and Kudrolli^14^ found that the distribution of the ridge lengths match a log-normal distribution. The same has been suggested by Wood^31^ and is supported by the statistical study of Sultan and Boudaoud^40^ on a minimal 1D model of a crumpled paper. Furthermore, Deboeuf *et al.*^7^ de-crumpled paper balls, after they were cut in half, to evaluate the number of layers. They scanned the de-crumpled paper to detect the ridge network and compared the obtained data on ridge lengths with a log-normal and a gamma distribution. They concluded that the gamma distribution overestimated the probability at small ridge lengths whereas the log-normal distribution showed a better fit. The log-normal distribution suggests the ridges will form in a hierarchical order.^7^ This is in line with the findings of Blair and Kudrolli^14^ according to which the ridges will randomly split during confinement, forming smaller ridges. Andresen and Hansen^12^ found that for the lower ridge lengths, the log-normal distribution fits well, supporting the findings of Blair and Kudrolli,^14^ Deboeuf *et al.*,^7^ and Sultan and Boudaoud.^40^ However, the distribution of the higher ridge lengths is better captured by a power-law function.^12^ Simulations of confining flexible flat sheets in a three-dimensional sphere^30^ support the finding that the distribution of ridge length could be described either by a log-normal or an exponential distribution.

Several theoretical studies state ridges always intersect at vertices.^33,46,47^ Research in hand-crumpled paper^12^ confirmed this statement. Andresen *et al.*^12^ observed that the maximum probability of the number of ridges intersecting at a vertex, also known as the node degree, was at the median, implying a Gaussian distribution. Only the tail of the distribution was better fitted by a log-normal distribution.^12^ Surprisingly, Blair and Kudrolli^14^ noticed that not all ridges are connected in flattened hand-crumpled paper. They investigated the number of neighbors at the end of a ridge and found that some ridges are without any neighbors. They provided two possible explanations for these findings. According to one of those explanations, this could be a consequence of the research method, as the ridges are examined after flattening the crumpled sheet of paper, which causes the recovery of elastic deformations. The alternative explanation is that the paper is capable of absorbing the forces producing the ridges, causing the ridges to dissipate near the ends due to the thickness of the sheet. During their study, they also discovered that a vertex with four ridges intersecting is the most common. Therefore, they looked at the angular distribution in a vertex where four ridges intersect. They found the angles are broadly distributed between 0° and 180° with predominant peaks at 20°, 60°, and 110°, which indicates that the d-cone geometry is dominant in a vertex where four ridges meet.^14^

Using the concept of node degree, Andresen and Hansen^12^ investigated the connectivity of different nodes and found that small-degree nodes tend to make a connection with large-degree nodes rather than other small-degree nodes. This is known as a disassortative network,^12,48^ which is found in nearly all technological and biological networks.^48^ The clustering coefficients that Andresen and Hansen^12^ found in their flattened hand-crumpled paper are in the range 0.13–0.23, indicating that the network is planar. In comparison with the clustering coefficient of a planar Delaunay network with similar spatial layout of the nodes, which describes the maximum possible clustering, the clustering coefficient in the crumpled paper is considerably lower. This shows that the ridge network in hand-crumpled paper does not develop highly interconnected sets of vertices.^12^

The vertices and ridges enclose a small area of the sheet called a facet.^12,13^ The number of ridges and vertices that enclose such a facet differ. In hand-crumpled paper, the facets were most often connected to three vertices^12^ and, thus, to three ridges. Furthermore, the distribution of the number of vertices per facet was best fitted by a log-normal function.^12^ Additionally, the facet sizes were found to match a log-normal distribution.^12^ Simulations on spherically crumpled elastoplastic sheets show a log-normal distribution of facet sizes.^13^ Tallinen *et al.*^13^ suggested that the average ridge length should scale with the size of a facet. Vliegenthart and Gompper^30^ simulated the crumpling of a flexible flat sheet in a sphere and found a log-normal distribution of the ridge length, corresponding well to the log-normal function found by Tallinen^13^*et al.* for the distribution of the facet area. However, Blair and Kudrolli^14^ found a slightly wider ridge length distribution in hand-crumpled paper. This could be due to the fact that one facet is surrounded by multiple ridges of various lengths.^13^ Interestingly, Tallinen *et al.*^13^ found that the distribution of facet area in elastic sheets was slightly better fitted by a gamma distribution, which was also observed for the ridge length distribution in a one-dimensional simulations of crumpling.^40^ The difference in facet size distribution in elastic and elastoplastic sheets could be explained by the difference in forming layers during crumpling, as elastic sheets show much stronger layering.^13^

Another interesting feature in crumpled materials is curvature. In crumpled materials, the facets contain low curvature, while the network of ridges contains high curvatures.^9^ Research on aluminum sheets crumpled in a cylindrical die has revealed that the curvature of ridges increases with the compaction ratio.^36^ Cambou and Menon^9^ studied the distribution of the radius of curvature in hand-crumpled aluminum and found that the radius of curvature at the peak of the histogram is much higher than what is required to initiate plastic folds in aluminum. This suggests that there is a limiting value of curvature at which new features are created by the crumpling process rather than increasing the curvature in the existing ridges. They also found a homogeneous distribution of radii of curvatures throughout the structure without any preference to originate at the confining walls. This is in contrast with what is found for crumpling in two dimensions by pulling a sheet through a hole and only look at the cross section, where the radii of curvature are different in the bulk of the material as compared to its boundaries.^9,49^

### 3.4. Energy storage

The energy used for crumpling a two-dimensional sheet into a three-dimensional structure is concentrated in the ridge network that arises during this process.^11,30,31,33,34,46^ Energy condensation occurs frequently in physics, and could for instance be observed in the gravitational collapse of galactic material, in fluid interfaces, and in encompassing turbulence.^31^ It has been shown that a ridge stores much more energy than a smoothly bent sheet of the same size.^31,46^ It has been therefore suggested that ridges are stronger than smoothly bent structures, because of the high energy content.^31,46^ Additionally, Wood^31^ concluded that the energy in vertices is substantially lower than the energy stored in ridges, which implies that the energetic properties of a crumpled sheet are defined by the ridges. Furthermore, he has shown that the ridges just weakly interact and that they are effectively independent. The energy (*E*) in a single ridge is known to scale as:^13,44^7
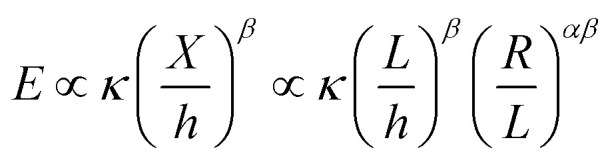
with *κ*, *X*, *h*, *R*, *L*, *α*, and *β* representing the bending modulus, the ridge length, the sheet thickness, the linear size of the sheet, and the scaling parameters, respectively. The scaling parameter *β* was found to be 1/3 for elastic sheets.^13^ With this knowledge, Wood^31^ derived an expression for the total energy (*E*_T_) in a crumpled structure:8
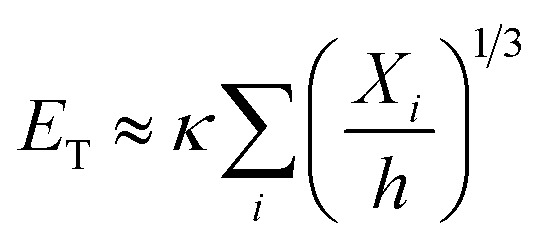


This approach was previously suggested by Lobovsky *et al.*^50^ in their simulations of crumpling elastic sheets in an impenetrable sphere. Tallinen *et al.*^13^ used a similar approach in their models of a rectangular elastic or elastoplastic sheet crumpled in a spherical shell. To find an expression for the total energy stored in a crumpled structure, they used an approximation for the number of ridges (*N*_X_):9
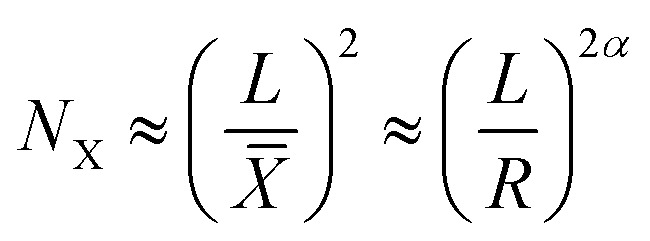
with *L*, *X̄*, *R* and *α* representing the linear size of the initial sheet, the average facet size, the radius of the crumpled ball, and a scaling parameter respectively, and multiplied this expression with the expression for the energy in a single ridge:10
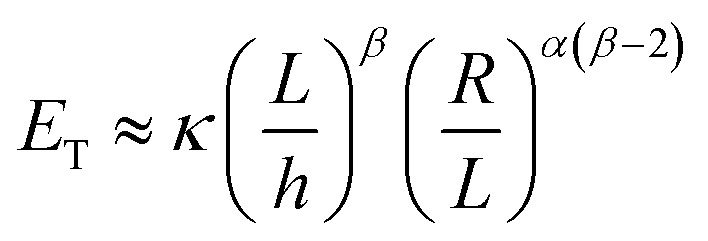


For elastic sheets they found *α* ≈ 1.65 for 
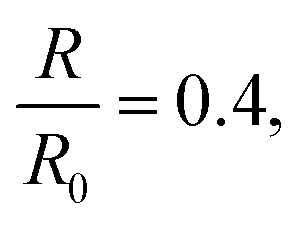
 where *R*_0_ is the initial shell radius and *β* = 1/3,^13^ corresponding to the previous statement. From this expression, a scaling relationship could be derived:

11 *E*^el^_T_ ∝ *R*^−2.76^

This scaling relationship for elastic sheets is equivalent to the findings of Vliegenthart *et al.*^30^ In elastoplastic sheets, ridges appear very early in the process because of plastic deformations. In the later phases of the crumpling process, the scaling of energy is similar to the elastic sheet. The scaling parameters are therefore similar as well.^13^

Another commonly used approach to determine the total energy stored in a crumpled sheet is to divide it into different energy contributions. Early research already suggested that the sum of stretching and bending energies is equal to the total energy.^50–52^ Chaïeb and Melo^51^ crumpled deoxidized high phosphorus (DHP) copper sheets in a cylindrical die and assumed the stretching energy to be constant, as the creases are self-similar and the stretching energy does not depend on the ridge length or the panel radius. Therefore, the only variable was the bending energy, which depends on the ridge length, rigidity, and the compaction ratio. Wood^31^ argued that the stretching energy is not constant, but that the ratio between the stretching and bending energy at the energetic minimum is equal to five. Vliegenthart and Gompper^30^ did simulations on a single ridge to predict the contributions of stretching and bending energy. They found a similar ratio of the stretching and bending energy on a single ridge when all sides of the sheets were fixed. However, when they fixed only one edge, the ratio was equal to twelve, which is probably caused by the relaxation of the elastic energy in the sheet.

Vliegenthart and Gompper^30^ state that in a flexible sheet, the Föppl-von Kármán number characterizes the elastic properties. They found an expression for the total energy (*E*_T_) in a crumpled sheet by integrating the force in eqn (3) with respect to the final radius:12
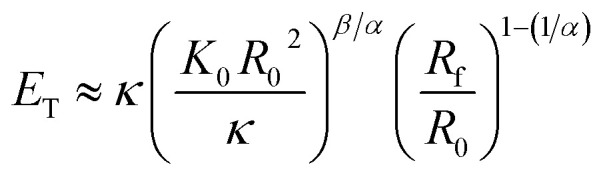
where *κ*, *K*_0_, *R*_0_, *R*_f_, *α* and *β* represent the bending rigidity, the two-dimensional Young's modulus, the radius of the flat sheet, the final radius of the crumpled ball, and the scaling parameters, respectively. For self-avoidant sheets, a value of 0.06 was found for *β* while 1 − (1/*α*) was found to equal −3, which resulted in an *α* value of 0.25. The first factor in this equation, *i.e.*
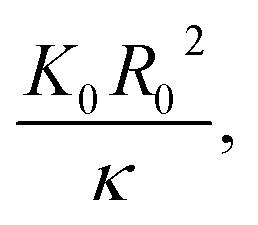
 represents the Fóppl-von Kármán number (*γ*). This expression is similar to the expression that Aharoni and Sharon^10^ found for the total bending energy (*E*_b_) in a temperature-responsive elastic gel, crumpled in a sphere:

13 *E*_b_ = *γ*^*α*^*η*^*β*^

where *η* represents the compaction ratio and *γ* stands for the Fóppl-von Kármán number, which in this case is constant. They found that the best fitted data was in agreement with the numerical simulations,^30,34^ with *α* = 1/6 and *β* = 3 ± 0.2.

## 4. Mechanical behavior

Thin crumpled membranes are lightweight structures without strong chemical bonds at mesoscale^37^ and exhibit a fascinating topology and an astonishing mechanical behavior.^6,7,27,38^ Their remarkable mechanical behavior is well reproducible^4^ and is a direct consequence of the energy stored in the ridge network created during the crumpling process as well as the internal contacts within the crumpled structure.^4,30,40,53,54^ The energy condensation in the ridges causes the crumpled structure to be highly resistant to compression.^53^ Additionally, it has been shown that a single ridge exhibits a buckling strength that is much higher than a smoothly bend membrane, with limited sensitivity to the distribution of the load over the ridge.^31,46^ To present an overview of the mechanical behavior of crumpled sheets, their strength, stiffness, Young's modulus, and Poisson's ratio will be addressed in this section, as a thorough understanding of these mechanical properties is essential to describing the stress–effective strain behavior in crumpled structures during axial compression.^37,38^

Furthermore, crumpled membranes are known to dilate after the compressing load is removed, quickly at first followed by a slow expansion.^36,53^ This is known as stress relaxation and is caused by the relaxation of the ridge network, which is created during the process of crumpling.^36^ This property could be very interesting in potential applications and will therefore be discussed at the end of this section.

### 4.1. Stress–strain behavior

**Fig. 3:**
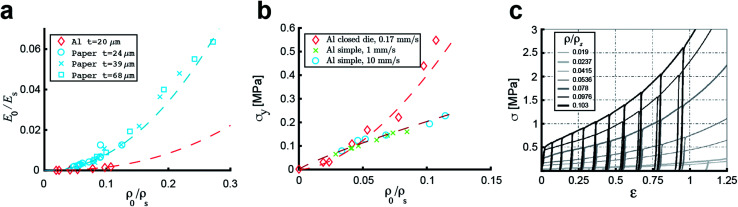
Mechanical behavior of a crumpled thin sheet. (a) The correlation between the initial relative modulus (*E*_0_/*E*_S_) and the initial relative density (*ρ*_0_/*ρ*_S_) for crumpled aluminum sheets (red), 20 μm thick, compressed at a compression rate of 0.17 mm s^−1^, fitted by eqn (14)^37^ and crumpled paper sheets (blue), 24, 39 and 68 μm thick, compressed at a compression rate of 0.1 mm s^−1^, which all could be fitted by eqn (15).^4^ (b) The yield stress (*σ*_y_) as a function of the initial relative density (*ρ*_0_/*ρ*_S_) for aluminum with thickness *t* = 20 μm compressed in closed die conditions at compression rate of 0.1 mm s^−1^ (red diamonds), fitted by eqn (16),^37^ as well as aluminum with thickness *t* = 22 μm compressed in a simple compression test at 1 (red crosses) and 10 mm s^−1^ (red circles) both fitted by eqn (17).^38^ (c) Stress–strain curves of crumpled aluminum samples with different initial relative densities, uniaxially loaded in a closed cylindrical die.^37^

Due to the concentration of energy within the ridge network created during the crumpling process, crumpled structures are highly resistant to compression.^4,53^ In particular, the resistance to hydrostatic compression is very high and much higher than the resistance during axial and sheer loading.^4,38^ Compression tests on crumpled aluminum sheets show elastoplastic behavior, in which the initial behavior is elastic and could be characterized by an initial Young's modulus.^36,37^ The initial relative modulus, the ratio of the modulus of the crumpled material (*E*_0_) to that of its bulk material (*E*_S_), has been shown to be dependent on the relative density, which represents the ratio of the density of the crumpled material (*ρ*_0_) to the density of its bulk material (*ρ*_S_) (Fig. 3a).^37,38^ Bouaziz *et al.*^37^ tested crumpled aluminum samples in closed die conditions and found the following relationship:14
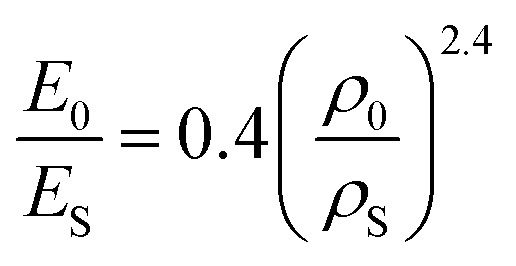


This result supports the conclusion of Balankin and Huerta^4^ that the relative Young's modulus of a crumpled material is related to its relative density through a power-law relationship. However, they found slightly different scaling parameters in their study in crumpled hyperelastic latex rubber and elastoplastic paper balls under axial compression using a simple compression test:15
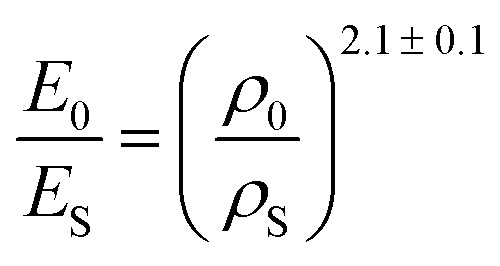


This difference could be caused by the differences in material or testing conditions. It has been shown that differences in the conditions of the compression tests will affect the strength of the crumpled material, as the required force to achieve a certain strain was higher in a compression test using closed die conditions than in a simple compression test.^36^ Research on crumpled graphene oxide (GO) presents a similar correlation between the relative density and the relative Young's modulus regardless of the fact that graphene structures could be bonded by van der Waals bonds or even be covalently bonded.^54^ This power-law relationship suggests that crumpled materials could be considered as a special class of cellular materials, as the same power-law dependence has been universally observed for cellular materials such as foams.^36,54–57^

In cellular materials, the yield stress (*σ*_y_) is also dependent on the relative density through a power-law function.^54–56^ The same has been observed for crumpled materials (Fig. 3b).^37,54^ Bouaziz *et al.*^37^ found that the yield stress (*σ*_y_) in hand-crumpled aluminum compressed under closed-die conditions scales as:16
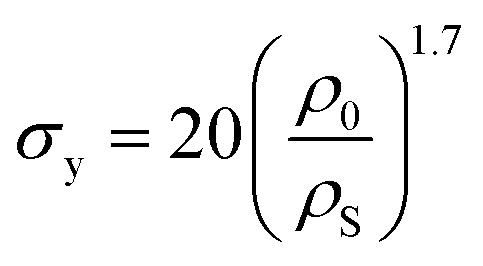


This suggests that the ridges and thin walls start to deform plastically.^37^ The yield stresses uncovered in simple compression tests of hand-crumpled aluminum are slightly lower than the ones found in a compression test under closed-die conditions^38^ and could be described by:17
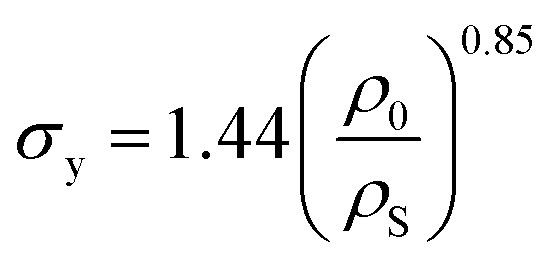


The transition from elastic to plastic behavior is clearly marked (Fig. 3c), as is the case in foams.^37^ However, when the yield stress (*σ*_y_) is reached, the stress (*σ*) within the material keeps increasing in contrast to foams that show a flat line.^37^ The plastic behavior after the yield stress is reached causes energy dissipation, leading to changes in the microstructure and hardening of the crumpled material.^36–38^ Strain hardening has been found in crumpled graphene oxide (GO) as well, and the hardening causes the material to become highly resistant towards further compaction both in dry conditions and solutions.^54^ Bouaziz *et al.*^37^ showed that in hand-crumpled aluminum, the increase in the stress (*σ*) after the yield stress has been reached, scales with densification:18
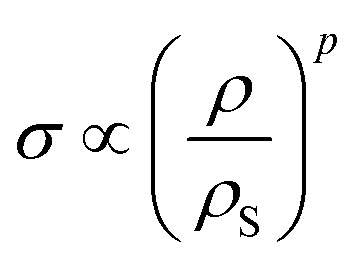


This relationship has been also found to hold for entangled materials.^37^ The values of the scaling parameter (*p*) are, however, lower in hand-crumpled aluminum (1.5 ≤ *p* ≤ 1.7) as compared to entangled materials (3 ≤ *p* ≤ 5).^37^

Cyclic loading at low strains shows a small hysteresis (Fig. 3c)^36–38^ with nonlinear recovery after unloading. However, the starting and finishing points of an unload-reload loop are very close and, once the loop is finished, the loading curve will continue along the initial monotonous loading curve.^37^ This small hysteresis indicates that the energy dissipation and internal friction are little,^36,38^ which is different from entangled wires.^36^ Possible explanations are a lower motility within the crumpled structure, more interlocking, and a limited topological rearrangement.^36,37^

The mechanical behavior does not solely depend on the internal properties such as the relative density of the crumpled material but may be also affected by a number of external factors such as the strain rate.^36^ It has been shown that crumpling aluminum foils in a cylindrical die using a high strain rate creates a material that exhibits lower stress values and, thus, lower strengths as compared to a sheet crumpled under a low strain rate.^36^ However, this effect was only observed in simple compression, while the effects of the strain rate were negligible in closed-die conditions.^36^ Additionally, the same study showed a decrease in the stiffness and strength when the aluminum foil was folded into two, four, or eight plies before crumpling, most likely because pre-folding causes the foil to organize and therefore decreases the internal friction in the crumpled structure.^36^ It was even shown that the conditions during the compression affected the strength of the crumpled material, as the required force to achieve a certain strain was higher in a compression test using closed-die conditions than in a simple compression test.^36^ Furthermore, Cottrino *et al.*^36^ studied the impact of several processing techniques on the strength and stiffness of crumpled aluminum. In the first group, a Gleeble machine was used to create bonding after crumpling the aluminum by applying an electric current, while the second group of samples were annealed after crumpling by keeping them in a salt-bath at 425 °C for 7 minutes. The samples of the last group were covered with a thin layer of epoxy glue at one side of the sheet before crumpling. Annealing the samples, group two, does not strengthen the crumpled material. However, the material is not substantially weakened by this procedure either, which indicates that the stiffness depends on the yield stress.^36^ The samples bonded using a Gleeble machine showed a minor increase in the stiffness and the strength of the crumpled aluminum.^36^ The glued samples show the highest increase in the mechanical behavior in crumpled aluminum due to the strong bonds that arise from the procedure.^36^

### 4.2. Poisson's ratio

During axial loading, materials experience lateral deformations without corresponding stresses. This phenomenon is known as the Poisson's effect based on which the Poisson's ratio (*ν*_e_) is defined as:^27^

**Fig. 4:**
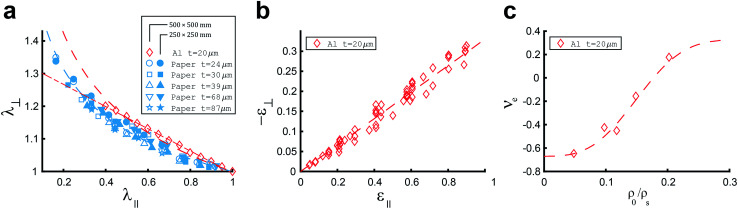
(a) Lateral expansion ratio (*λ*_⊥_) as a function of axial compression ratio (*λ*_∥_) for crumpled aluminium sheets (red diamonds) of initial size 500 × 500 mm, fitted by eqn (20) with *ν* = 0.22 (red dashed line) and eqn (19) with *ν*_e_ = 0.34 (red dash-dot line), and for crumpled paper sheets (blue symbols) of initial size 500 × 500 mm (open symbols) and 250 × 250 mm (closed symbols), fitted by eqn (20) with *ν* = 0.17 (blue dashed line). (b) Lateral strains (−*ε*_⊥_) *versus* axial strains (*ε*_∥_) for crumpled aluminum sheets (red diamonds) of thickness *t* = 20 μm and initial size 500 × 500 mm and 200 × 200 mm, approximated by eqn (19) with *ν*_e_ = 0.33 (red dashed line).^27^ (c) The correlation between the Poisson ratio (*ν*_e_) and the relative density in crumpled aluminum sheets of thickness *t* = 20 μm and different initial sizes to create different relative densities, fitted by *ν*_e_ = 0.33 − exp(−200(*ρ*_0_/*ρ*_S_)^3^).^37^

19 *ν*_e_ = −*ε*_⊥_/*ε*_∥_

where *ε*_⊥_ and *ε*_∥_ are the strain in lateral and axial directions, respectively. In general, the Poisson's ratio could be affected by the structure, porosity, and chemical composition of the material.^27^ The Poisson's effect in some materials could be expressed as a power law:

20 *λ*_⊥_ = *λ*_∥_^−*ν*^

where *λ*_⊥_, *λ*_∥_ and *ν* represent the lateral and axial compression/expansion and the Poisson's index, respectively. Huerta *et al.*^53^ studied randomly folded thin materials and found a universal Poisson's index of *ν* = 0.17 ± 0.01. This value was found in crumpled elastoplastic paper sheets as well, in both axial and radial compression.^27^ Research in hand-crumpled paper suggests that the Poisson's ratio does not depend on environmental conditions, contraction ratio, sheet size, or paper thickness (Fig. 4a).^27^

Gomes *et al.*^58^ found that, in crumpled aluminum foils, the Poisson's indices depend on the foil thicknesses. However, Balankin *et al.*^27^ showed that the Poisson's effect in crumpled aluminum foil could be captured by a universal Poisson's index of *ν* = 0.25 ± 0.04, although a universal linear relationship (Fig. 4a and b) with a Poisson's ratio of *ν*_e_ = 0.33 ± 0.02 showed even a better approximation. The difference in the dependence of the Poisson's effect for plastic and elastoplastic sheets suggests that they belong to different universality classes.^27^

Bouaziz *et al.*^37^ evaluated the transverse compression Poisson's ratio upon unloading as well. Those observations showed the Poisson's ratio varies with the relative density (*ρ*_0_/*ρ*_S_) of the crumpled material.^37^Fig. 4c shows that below a relative density of 0.15, in the transverse process of compression, the Poisson's ratio is negative,^37^ which signifies that the material is auxetic.^27^ This kind of auxetic behavior could make crumpling an even more interesting basis for creating mechanical metamaterials, as the auxetic behavior^59^ is one of the most sought after behaviors when designing mechanical metamaterials.

### 4.3. Relaxation

**Fig. 5:**
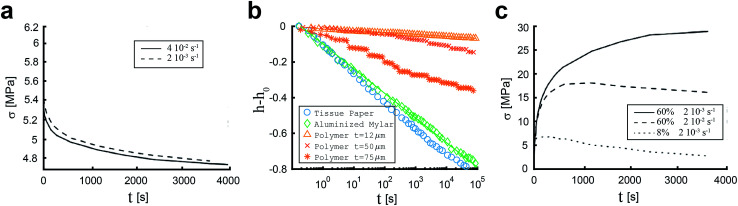
(a) Stress relaxation after loading a crumpled aluminum sheet of thickness *t* = 14 μm and initial size 280 × 280 mm, strained at 2 × 10^−2^ s^−1^ (dashed line) and 4 × 10^−2^ s^−1^(solid line).^36^ (b) Relaxation after loading by keeping the load constant (200 g) and observing the change in height (*h* − *h*_0_) in tissue paper (Kimwipes®: one-ply white wipers 381 × 431 mm),^11^ aluminized mylar sheets, circular with *D* = 340 mm and *t* = 12.5 μm, fitted by *h* − *h*_0_ = −0.14 log(*t*)^11^ and Melinex polymer foils, 210 × 210 mm and thickness variating between 12 and 75 μm.^45^ (c) Stress relaxation after discharging the load on a crumpled aluminum sheet of thickness *t* = 14 μm and initial size 280 × 280 mm of a sample strained at 2 × 10^−3^ s^−1^ with volume fraction 60% (solid line), a sample strained at 2 × 10^−2^ s^−1^ with volume fraction 60% (dashed line) and a sample strained at 2 × 10^−3^ s^−1^ with volume fraction 8% (dashed line).^36^

Crumpled materials tend to uncrumple when the load is released, first rapidly and then the crumpled material will slowly increase in size for up to days.^36^ This is due to the relaxation of the ridge network that is created during the crumpling process.^36^ Relaxation could be studied after discharge of the load, but it could be also studied by keeping the displacement constant and measuring the corresponding stresses.^29,36,38,53^ Measured relaxation after loading shows a similar pattern with a fast relaxation at first followed by a slow relaxation.^36,38,53^ Cottrino *et al.*^36^ investigated the relaxation after loading in crumpled aluminum foils and found that the fast relaxation could be a consequence of the relaxation of the aluminum foil. The slow relaxation, however, was attributed to the relaxation of the ridge network. Multiple studies^26,36,38,53^ have shown that an exponential function fits the stress relaxation data the best:

21 *F*(*t*) = *F*(0)exp[−(*t*/*τ*)^*β*^]

where, *F*(*t*), *F*(0), *t*, *τ* and *β* represent the force at time *t*, the force at *t* = 0, the time, the characteristic time, and a scaling parameter, respectively. Eqn (21) covers stress relaxation over a large span of time (over 10^3^ s). The stress relaxation appears to be independent of the edge sizes, the geometry of the sheet, the compression ratio, or the initial packing density.^36^ However, the stress relaxation tends to increase with strain rate (Fig. 5a), which could be explained by the fact that the structure will rearrange more when subjected to a high strain rate, causing a higher relaxation.^36^ The stress at relaxation will reach a finite saturation value, which is not zero, suggesting that crumpled materials could be classified as a solid structure.

Instead of keeping the displacement constant, the compression force could be kept constant to study the creep behavior of crumpled sheets. This method was used by Matan *et al.*^11^ as they placed a mass on the piston that compressed aluminized Mylar in a cylindrical die. They studied the changes in the height during a couple of weeks and observed that the asymptotic height was not reached even after three weeks. They found a logarithmic dependence of the height on time:

22 *h* = *a* − *b* log(*t*/s)

where *h* and *t* represent the height of the piston and time. Parameters *a* and *b* are the scaling parameters. This logarithmic dependence (Fig. 5b) implies energy dissipation, which could be caused by plastic flow in areas with high curvature or by friction.^11^ Additionally, Matan *et al.*^11^ showed that tissue paper exhibits a relaxation behavior that is similar to Mylar, but Cotton balls presented a smaller relaxation during several weeks. Dierking and Archer^45^ found the same logarithmic dependence in crumpled polymer films (Fig. 5b). They observed that the relaxation of polymer films depends on the thickness of the film, as thicker films exhibited more sudden discontinuous stress relief. That is most likely caused by the lower ridge density in thicker films.^45^

When measuring the relaxation after the discharge of the confinement load, the increase in ball diameter is usually measured. Huerta *et al.*^53^ found that, during a ten days period, the increase in the ball diameter of elastoplastic sheets scales as:

23 *R*(*t*) = *R*(0) + *ν*(*R*/*L*)ln(*t*/*τ*_ε_)

where *R*(0), *R*, *L*, *ν*, *t* and *τ*_ε_ are the initial ball radius, characteristic ball size, edge size of the initial flat square sheet, strain relaxation rate, time, and characteristic time, respectively. In contrast to stress relaxation after loading, it was noticed that the relaxation is more significant for lower strain rates as compared to higher strain rates.^36^ The results of the relaxation of crumpled aluminum sheets after discharge of the confinement force (Fig. 5c) also suggest a dependence on the loading rate.^36^

## 5. Potential and applications

**Fig. 6:**
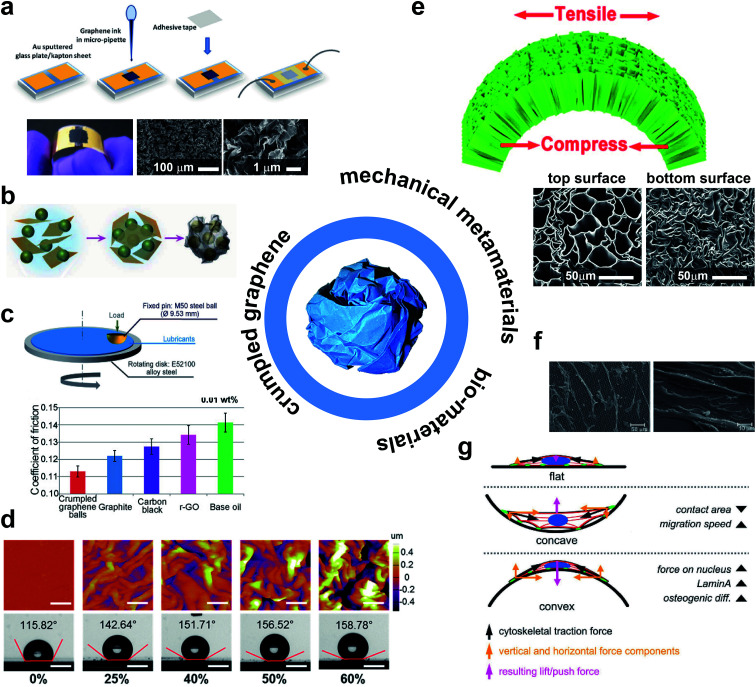
An overview of potential applications for crumpled-based materials. (a) Robust sensation of strain and pressure using crumped sheets of graphene oxide.^60^ (b) Encapsulation of Si nano-particles using graphene micro-meter sized graphene sheets through the evaporation process of liquid droplets to improve the performance of lithium ion batteries.^61^ (c) Reduction of friction coefficient of lubricants using crumpled graphene balls.^62^ (d) Dynamic control of surface wettability based on crumpled patterns of molybdenum disulfide (MoS_2_) on a polystyrene (PS) substrate achieved *via* shrinkage of PS layer.^63^ (e) Super flexibility achieved based on a simple chemical treatment of natural wood.^66^ The micro structure of super flexible wood might be mimicked artificially based on crumpling of planar materials. (f) The surface of crumpled-based scaffolds can be functionalized to adjust the pattern of cell adhesion.^69^ (g) Surface curvature can adjust the cytoskeletal tensions.^77^

Crumpling a thin, 2D sheet into a 3D structure could be used to add functionalities to existing materials. Because of the flexibility in stiffness, strength, porosity, and many other properties, a wide range of properties may be obtained and, as a result, numerous potential applications could be envisaged. Several potential applications have been previously mentioned in literature. An overview of potential applications is given in Fig. 6.

Crumpled graphene may be promising for creating new ways to store energy with applications in sensors, actuators, and solar cells.^54^ Crumpled graphene oxide (GO) has been used to create strain and pressure sensors with extremely high pressure sensing capability at ultra-small strains (Fig. 6a).^60^ Moreover, microscale graphene oxide (GO) sheets have been used to encapsulate Si nano-particles based on capillary forces during evaporation of aerosol droplets (Fig. 6b).^61^ Composite capsules of GO/Si can greatly improve the performance of lithium ion batteries. Miniaturized crumpled graphene balls have been shown to be highly influential in reduction of friction and wear while used as additives in lubricant-based oils (Fig. 6c).^62^ The 2D crumpling of molybdenum disulfide (MoS_2_), based on shrinkage of a polystyrene (PS) substrate, has been shown as a dynamic way to tune the level of wettability (Fig. 6d).^63^ Furthermore, crumpled materials have been shown to combine the mechanical behaviors of foams, a clearly marked elastic to plastic transition, and entangled fibrous materials, strain hardening after the yield stress is reached,^37,64^ which makes them a suitable candidate for numerous engineering applications such as lightweight sandwich panels, shock absorbers, or as a mechanical metamaterial for mechanical cloaking.^6,36,38^ Additionally, crumpled materials are a feasible alternative to more expensive engineering materials such as cellular metals due to their relatively easy production process. While maintaining robust mechanical properties^17,36,54,65^ they may provide super flexibility due to their cellular design (Fig. 6e).^66^

Crumpled materials could also be of interest for medical applications.^54^ Given their rich physics and the unusual combination of properties that they offer, crumpled sheets could be also used as a basis for creating advanced functionalities.^59^ For example, Bouaziz *et al.*^37^ showed that the crumpled material could be designed in such a way to exhibit auxetic behavior, meaning that the material would expand in the lateral direction when it is stretched, instead of contracting.^59^ Auxetic behavior has been found in several natural systems such as cancellous bone, tendons, living cow skin, certain zeolites, and some minerals.^59^ In addition to the unusual mechanical response of auxetic materials, the possibility to attain unusually high values of other material properties such as hardness, fracture toughness, indentation resistance, and energy absorption makes them very interesting.^59^ Auxetic materials could therefore be appealing for creating stents and smart bandages.^59^ Furthermore, they are highly interesting for orthopedic implants, as they could be designed to mimic cancellous bone and to fabricate meta-implants^59^ that lead to a longer implant longevity.

With the use of surface treatment techniques, crumpled structures could be also used to improve tissue regeneration within the context of what is referred to as meta-biomaterials^17^ in the literature. Most conventional techniques for surface enhancement are only applicable to flat surfaces and are therefore not useful for three-dimensional scaffolds.^18–20^ As a crumpled material initially starts from a two-dimensional state, the entire surface area is accessible and could be functionalized using conventional techniques, thereby allowing for fabrication of meta-biomaterials that combine complex three-dimensional shapes with surface-related functionalities.^20,67^ It has been shown that chemical and/or topological cues at the surface could significantly influence the behavior of cells and regulate cell orientation, cell morphology, cell proliferation, and cell differentiation (Fig. 6f and g).^68–72^ Additionally, the same type of surface-related cues, *e.g.* surface nanopatterns, could also affect the growth of bacteria^73^ by preventing bacteria from attaching to the surface of biomaterials and forming a biofilm,^68^ which may be an alternative for conventional antimicrobial agents or disinfectants, leaving toxic residue.^74^ Another advantage of starting from a flat surface is the possibility to incorporate drug delivery vehicles, which could then be used to deliver therapeutic agents to a specific part of the body.^22,75,76^

After surface functionalization, a three-dimensional structure could be obtained through crumpling. There are other techniques known for transforming a two-dimensional structure into a three-dimensional one including origami-based techniques.^21,22^ Most of those techniques result in an ordered origami arrangement, which are susceptible to defects.^17,54^ Relatively small imperfections could substantially affect the mechanical properties of the ordered three-dimensional construct.^17,54^ However, crumpling results in a disordered arrangement, which is less sensitive to imperfections.^17^ To use the crumpled structure for tissue regeneration, the structure should be porous to ensure cell nutrition, oxygenation and migration.^17^ In addition, the complex morphological design of crumpled-based materials can be beneficial to adjust the attachment of cells to the crumpled substrate (Fig. 6g), cell migration and differentiation of stem cells based on the patterns of forces acting on their nucleus.^77^ Mirzaali *et al.*^17^ studied the effects of circular holes created in crumpled Mylar sheets and concluded that the crumpling behavior is generally similar to that of sheets without holes. However, the crumpling exponents were somewhat higher, indicating that the rate of increase in the crumpling force is higher for porous sheets, which was explained by the fact that the edges of the holes could interlock. An important factor for the increase of the crumpling exponents was the fraction of surface occupied by the holes: the higher this fraction, the higher the increase of the crumpling exponent.^17^

In conclusion, crumpled structures are promising candidates for applications in multiple fields, due to the unusual mechanical properties of crumpled matter and the possibility to tune those properties. The fact that crumpling starts from a flat sheet increase the possibilities even further particularly in the areas where surface-related functionalities are of interest.

## Conflicts of interest

There are no conflicts to declare.

## Supplementary Material
